# Request of endocrinology and metabolism journals for data sharing statements in clinical trial reports: a survey

**DOI:** 10.3389/fmed.2025.1518399

**Published:** 2025-05-21

**Authors:** Yingxin Liu, Bo Chen, Jingyi Zhang, Xuerui Bai, Lili Kang, Wenli Li, Guowei Li

**Affiliations:** ^1^Center for Clinical Epidemiology and Methodology (CCEM), The Affiliated Guangdong Second Provincial General Hospital of Jinan University, Guangzhou, China; ^2^Department of Endocrinology, The Affiliated Guangdong Second Provincial General Hospital of Jinan University, Guangzhou, China; ^3^Department of Epidemiology, School of Medicine, Jinan University, Guangzhou, China; ^4^School of Public Health, Guangdong Pharmaceutical University, Guangzhou, China; ^5^Department of Infectious Diseases, The Affiliated Guangdong Second Provincial General Hospital of Jinan University, Guangzhou, China; ^6^Father Sean O’Sullivan Research Centre, St. Joseph’s Healthcare Hamilton, Hamilton, ON, Canada

**Keywords:** data sharing, clinical trial, ICMJE, endocrinology, metabolism

## Abstract

**Background:**

To enhance reproducibility and transparency, the International Committee of Medical Journal Editors (ICMJE) required that all trial reports submitted after July 2018 must include a data sharing statement (DSS). Accordingly, emerging biomedical journals required trial authors to include a DSS in submissions for publication if trial reports were accepted. Nevertheless, it was unclear whether endocrinology and metabolism journals had this request for DSS of clinical trial reports. Therefore, we aimed to explore whether endocrinology and metabolism journals requested DSS in clinical trial submissions, and their compliance with the declared request in published trial reports.

**Methods:**

Journals that were from the category of “Endocrinology & Metabolism” defined by Journal Citation Reports (JCR, as of June 2023) and published clinical trial reports between 2019 and 2022, were included for analysis. The primary outcome was whether a journal explicitly requested a DSS in its manuscript submission instructions for clinical trials, which was extracted and verified in December 2023. We also evaluated whether these journals indeed included a DSS in their published trial reports that were published between December 2023 and May 2024.

**Results:**

A total of 141 endocrinology and metabolism journals were included for analysis, among which 125 (88.7%) requested DSS in clinical trial submissions. Journals requesting DSS had a significantly lower JCR quartile and higher impact factor when compared with those journals without DSS request. Among the 90 journals requesting DSS, 14 (15.6%) journals indeed did not publish any DSS in their published trial reports between December 2023 and May 2024.

**Conclusion:**

Over 10% of endocrinology and metabolism journals did not request DSS in clinical trial submissions. More than 15% of the journals declaring to request DSS from their submission instructions, did not publish DSS in their published trial reports. More efforts are needed to improve the practice of endocrinology and metabolism journals in requesting and publishing DSS of clinical trial reports.

## Introduction

Sharing individual participant data (IPD) can enhance scientific progress, promote transparency and integrity, advance research collaboration, and increase the generalizability of findings (1, 2). Data sharing in clinical trials in endocrinology and metabolism is important, especially given the rapid increase in global disease burden of type 2 diabetes mellitus (3–5). During the period from 2018 to 2023, clinical trials of endocrine and metabolic disorders accounted for approximately 6% of all registered trials globally, ranking in fourth place among all the therapeutic areas (6). To enhance data sharing in clinical trials, the 2017 update to the International Committee of Medical Journal Editors (ICMJE) recommendations included a recommendation that, effective from July 2018, all publications of clinical trials should include a data sharing statement (DSS) (7).

Providing a DSS is reported to help increase the actual data sharing, and improve the reproducibility and reporting quality (8, 9). Hardwicke et al. (10) found an improvement in data sharing from 22 to 62% after the implementation of journal request for DSS. Since the ICMJE requirement, emerging biomedical journals required trial authors to include a DSS in submissions for publication if trial reports were accepted, which had led to inclusion of a DSS becoming a norm in the literature (11). Some previous studies investigated journal request for DSS in various clinical fields, yet none focused on endocrinology and metabolism journals (12–20). Thus, little was known whether endocrinology and metabolism journals had this request for DSS of clinical trial reports. Likewise, the actual publication of DSS in trial reports remained unexplored, especially for those journals clearly claiming to request DSS in their manuscript submission instructions.

Therefore, we conducted this survey to explore the current practice of endocrinology and metabolism journals for requesting and publishing DSS in clinical trials.

## Materials and methods

### Inclusion and exclusion criteria

**Figure 1:**
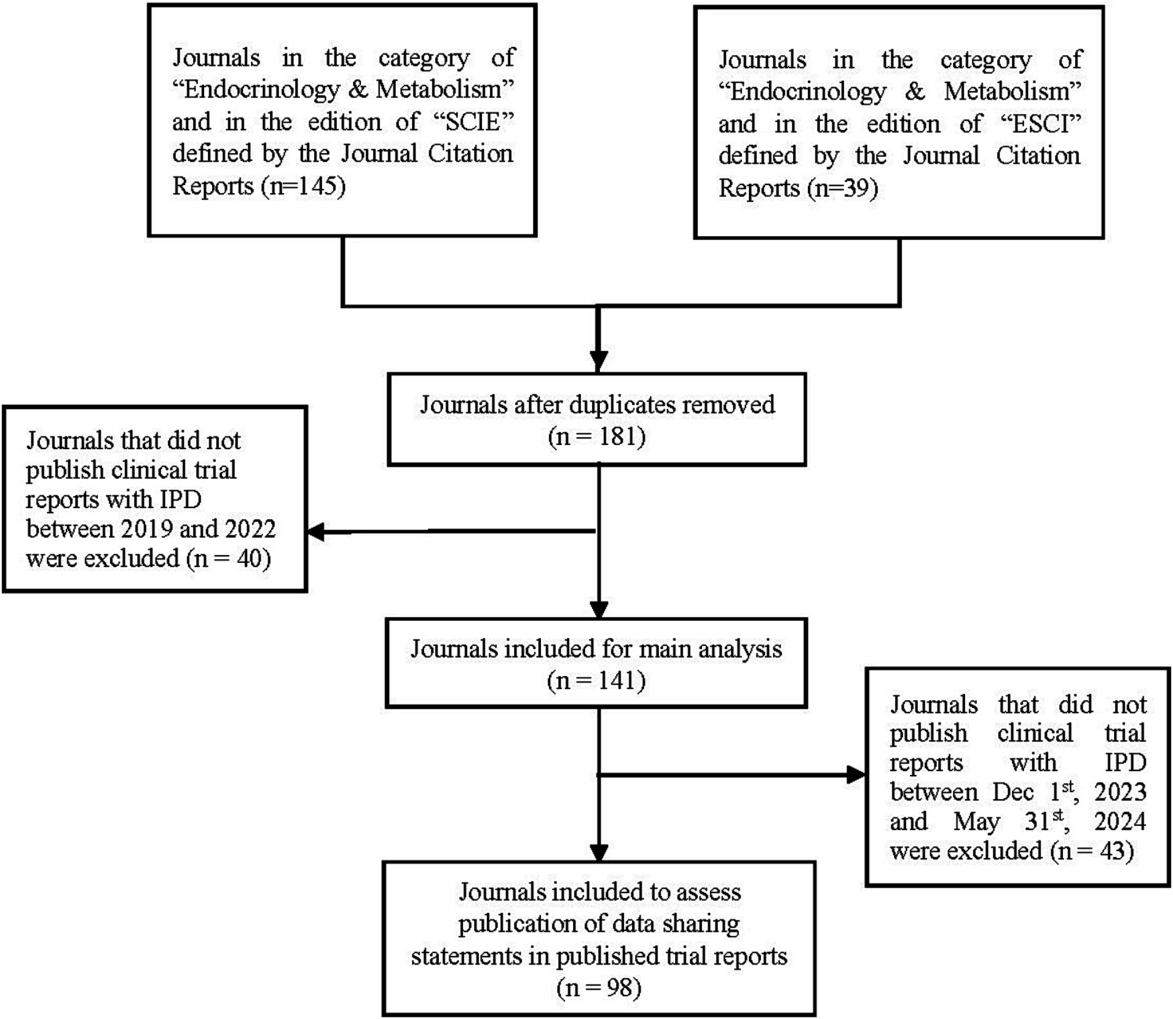
Flow diagram showing journal selection process for this study.

We first selected all 181 journals that were from the category of “Endocrinology & Metabolism” defined by Journal Citation Reports (JCR, as of June 2023). Given the ICMJE requirement for clinical trials submitted after July 2018, only those journals that published trial reports between 2019 and 2022 were eligible for inclusion. The inclusion from January 2019, was determined to align with the ICMJE recommendation (effective from July 2018). This half-year adaptation window allowed sufficient time for journals to initiate and update their request for DSS in trials, which was consistent with previous studies (14, 21). We therefore excluded 40 journals that did not publish any trial reports with IPD during that time period after comprehensively searching PubMed and journals’ webpages using the keywords of “trial,” “clinical trial,” “RCT,” “intervention,” “interventional”, and “phase.” A total of 141 endocrinology and metabolism journals were included for analysis (Figure 1). We also added Supplementary Figure 1 to show the timeframe of this study for clarity.

The Strengthening the Reporting of Observational Studies in Epidemiology (STROBE) guideline was used to report this survey (22–24).

### Study outcome

The primary outcome was whether a journal explicitly requested a DSS in its manuscript submission instructions for clinical trials in the website or in the submission portal (*Yes* or *No*). This outcome was extracted and verified in December 2023 (Supplementary Figure 1). A journal was deemed to request a DSS if it clearly asked trial authors to provide a DSS in submission along with their trial reports, or in the separate section entitled “data sharing,” “data availability,” “data accessibility,” “research data,” “data deposit” or “data deposition.” We used Google Translate to translate journals’ submission instructions into English for those journals that were not in English or Chinese language.

Some journals declared to follow ICMJE requirement, but did not explicitly request DSS for clinical trials in their submission instructions. To assess whether these journals actually requested DSS, we performed mock submissions to evaluate their submission systems. Additionally, we identified journals that asked authors to upload a statement or ask authors about their willingness to share data in submission systems as having a DSS request. For those journals without DSS request in submission instructions, our mock submissions confirmed that none requested DSS in their submission systems.

We further categorized journals based on their request strength for DSS. From their manuscript submission instructions, the journal request strength was grouped as *Weak* if the terms “encourage” or “recommend” were used (e.g., *authors are encouraged to provide a data sharing statement*). Journals were grouped as having *Strong* request if they used the terms “mandate,” “must,” “require,” “request” or “should” (e.g., *all research articles should include a data sharing statement*). These terms used to classify the strength of journal request for DSS were mainly adopted from previous research (13, 25). Therefore, there were three journal groups (having no request, weak request, and strong request) for supplemental analysis.

### Data extraction

All information was extracted by authors independently from December 2023 to May 2024 (YL, JZ, and XB). Disagreement was solved by consensus among the authors or by consulting a senior investigator (GL).

Data on journal characteristics were extracted including the percentage of open access, publisher, publication language, journal impact factor in 2022 (released in June 2023), JCR quartile, the total number of trials published between 2019 and 2022, whether the journal was on the ICMJE list in the official website (26), whether the journal explicitly endorsed the Consolidated Standards of Reporting Trials (CONSORT) in its submission instruction, gender of journals’ editor-in-chief, and region of editor-in-chief’s institution. The percentage of open access denoted the percentage of open access items among all the citable items published in the past 3 years, as extracted from the open access section in the JCR. For journals with more than one editors-in-chief, we only selected the first one presented on the journal webpages to avert double counting. Also, because there were no multiple journals sharing the same editors-in-chief, no duplicate editors-in-chief were selected for our analysis.

### Statistical analysis

We described continuous characteristics using medians with lower and upper quartiles (Q1–Q3), and categorical variables using counts and percentages. Comparisons of journal characteristics between journals with and without DSS request were conducted by Wilcoxon rank sum test and chi-square test for continuous and categorical variables, respectively. Comparisons between the three journal groups (having no request, weak request, and strong request) were performed by Kruskal–Wallis test (for continuous variables) and chi-square test (for categorical variables) respectively.

An exploratory analysis was conducted to explore the potential temporal trend of journal request for DSS. We first matched our included journals with two previous studies in 2018 that had the largest numbers of medical journals and investigated journal request for DSS (13, 14). Specifically, one study included 503 biomedical journals (14) and the other included 700 journals covering the fields of physical sciences, life and health (13). Difference in the proportion of endocrinology and metabolism journals having request for DSS was evaluated as the change from 2018 to 2023.

To assess the potential discordance between journals’ declared request for DSS (from their manuscript submission instructions) and actual publication of DSS (from their published trial reports), we first located journals that had published any trial reports with IPD between December 2023 and May 2024 (Supplementary Figure 1). We determined whether these journals published any DSS in their trial reports by thoroughly searching the main manuscripts, the supplementary materials, and the webpages in which the journals published these reports. Journals were therefore identified to publish any DSS in their published trial reports (*Yes* or *No*). Subsequently, McNemar’s test was used to examine whether there was a significant discordance between journals’ declared request and journals’ actual published DSS. Additionally, the difference in the proportions of journals publishing DSS in clinical trials between the three journal groups (having no request, weak request, and strong request) was performed by chi-square test.

All statistical tests were two-sided with a significance level of 0.05. Analyses were conducted in R software version 4.4.0.

## Results

A total of 141 endocrinology and metabolism journals were included for analysis (Supplementary Table 1 shows the list of included journals). As shown in Table 1, the journals were mainly published in English language (98.6%) and had a male editor-in-chief (80.1%). The median open access percentage was 24.5% (Q1–Q3: 11.1–93.5) and impact factor 2.9 (1.9–4.5). There were 31.9% of the journals on the ICMJE list and 58.2% endorsing CONSORT. The journals published a median of 11 trials between 2019 and 2022 (Q1–Q3: 5.0–26.8). Elsevier, Springer, and Wiley were ranked the top three publishers with the largest number of journals (Figure 1; Supplementary Figure 2). The journal editors-in-chief were mainly from the USA (32.6%), UK (12.8%), Italy (10.6%) and Germany (7.1%) (Supplementary Figure 3).

**Table 1:** Descriptions of journal characteristics for the 141 included journals^a^.

Journal characteristics	Overall (*n* = 141)
Open access percentage: median (Q1–Q3)	24.5 (11.1–93.5)
Open access percentage ≥50%	
No	92 (65.2)
Yes	49 (34.8)
Publisher	
Elsevier	26 (18.4)
Springer	21 (14.9)
Wiley	18 (12.8)
Others	76 (53.9)
Publication language	
English	139 (98.6)
Non-English	2 (1.4)
Journal impact factor: median (Q1–Q3)	3.5 (2.5–4.9)
Journal impact factor ≥3.5^b^	
No	68 (48.2)
Yes	73 (51.8)
JCR quartile	
Q1–Q2	67 (47.5)
Q3–Q4	74 (52.5)
Whether the journal was on the ICMJE list	
No	96 (68.1)
Yes	45 (31.9)
Whether the journal endorsed CONSORT	
No	59 (41.8)
Yes	82 (58.2)
Number of trials published between 2019 and 2022: median (Q1–Q3)	18 (5–38)
Number of trials published between 2019 and 2022 ≥18^b^	
No	70 (49.6)
Yes	71 (50.4)
Gender of editor-in chief	
Female	28 (19.9)
Male	113 (80.1)
Region of the institution of editor-in chief	
USA	46 (32.6)
UK	18 (12.8)
Italy	15 (10.6)
Germany	10 (7.1)
Others	52 (36.9)

JCR, Journal Citation Reports; ICMJE, International Committee of Medical Journal Editors; CONSORT, Consolidated Standards of Reporting Trials; Q1, first quartile; Q3, third quartile.

a Results shown as count (%) unless otherwise specified.

b The median Journal Impact Factor was 3.5; median number of trials published between 2019 and 2022 was 18.

**Figure 2:**
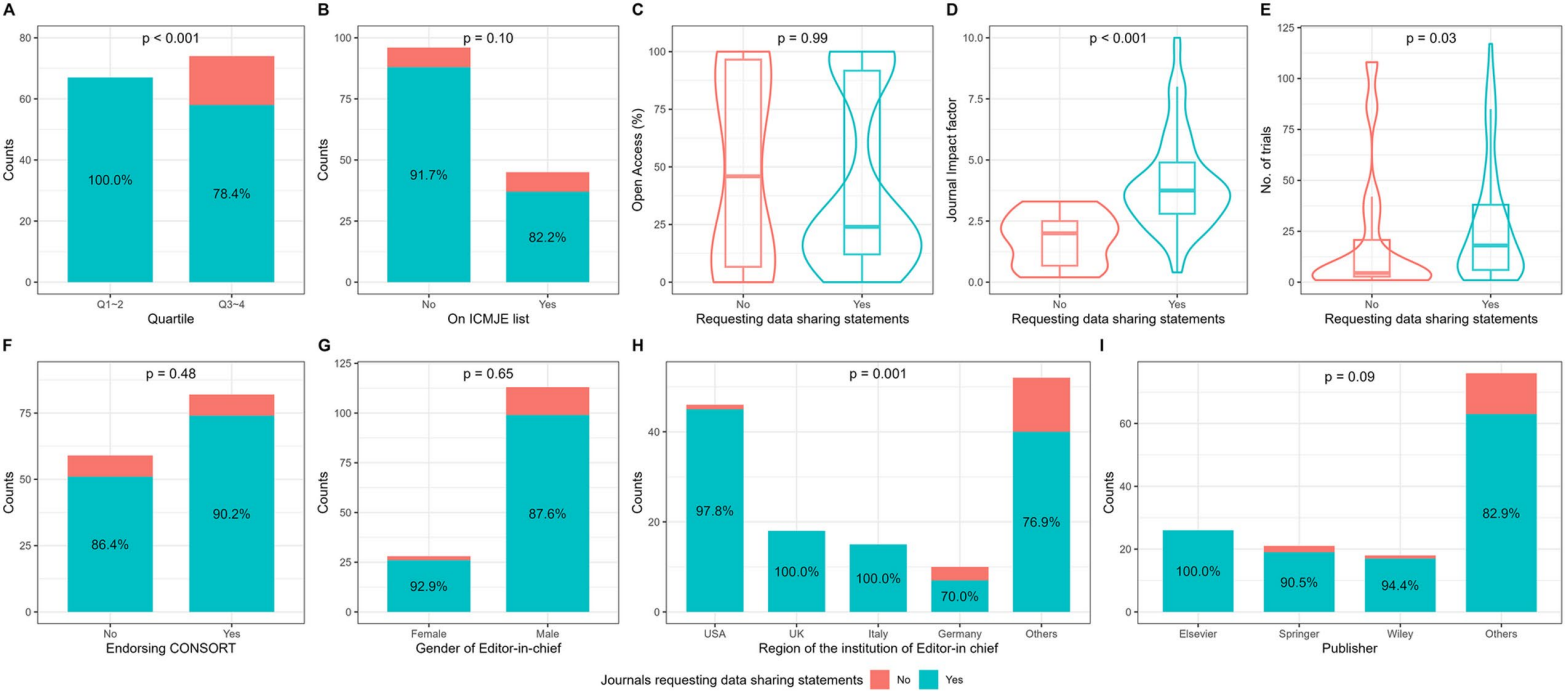
Comparisons of journals with and without data sharing statement request. By **(A)** JCR quartile, **(B)** whether the journal was on the ICMJE list in the official website, **(C)** the percentage of Open Access, **(D)** journal impact factor, **(E)** the total number of trials published between 2019 and 2022, **(F)** whether the journal explicitly endorsed CONSORT in submission instruction, **(G)** gender of journals’ editor-in-chief, **(H)** region of editor-in-chief’s institution, **(I)** publisher.

There were 125 (88.7%) journals requesting DSS in clinical trial submissions. As presented in Figure 2, journals requesting DSS were more likely to have a lower JCR quartile, higher impact factor and larger number of published trials (*p* < 0.05). Significant difference in the proportion of journals requesting DSS was found between regions of editor-in-chief’s institutions. Among journals requesting DSS, 51 (40.8%) had weak request and 74 (59.2%) strong request. As shown in Supplementary Table 2, significant differences in open access percentage, publisher, impact factor, JCR quartile, proportion of endorsing CONSORT, and region of editor-in-chief’s institutions were found among the three journal groups (having no request, weak request, and strong request).

**Figure 3:**
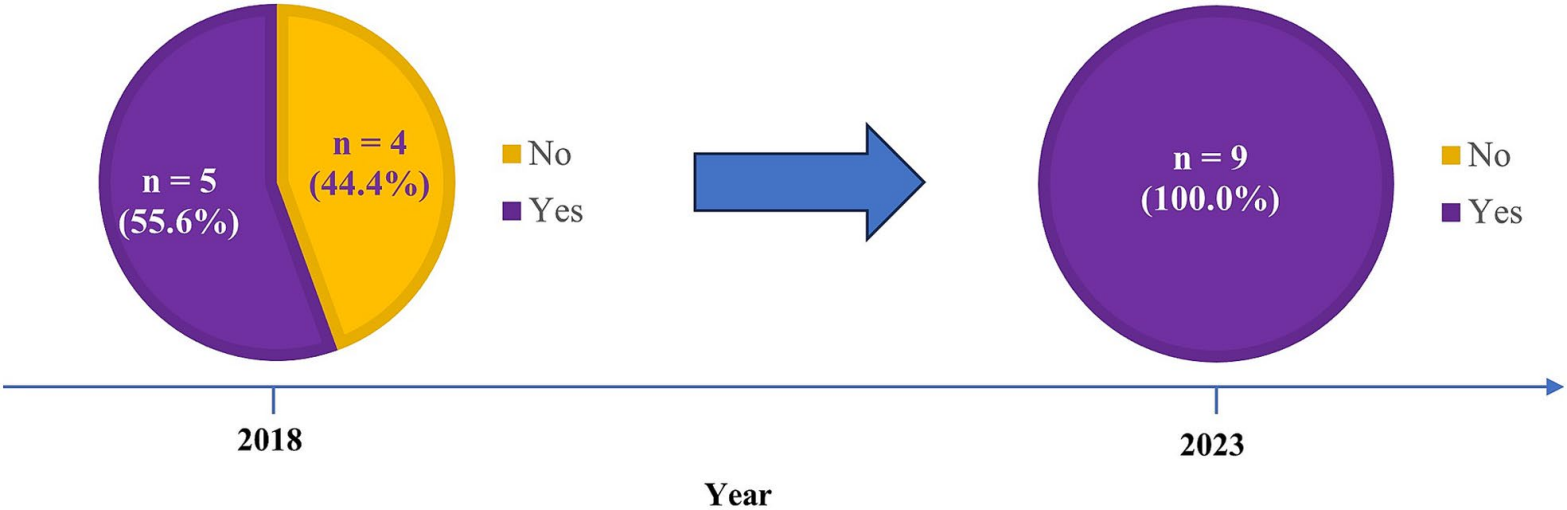
Exploratory analysis results from comparing previous studies published in BMJ Open and PeerJ with our current study regarding the journals requesting data sharing statements.

There were nine journals that were included in both previous studies and our survey (Figure 3). Four journals (44.4%) that did not request DSS as identified in 2018, were found to change to have DSS request from our current survey.

**Figure 4:**
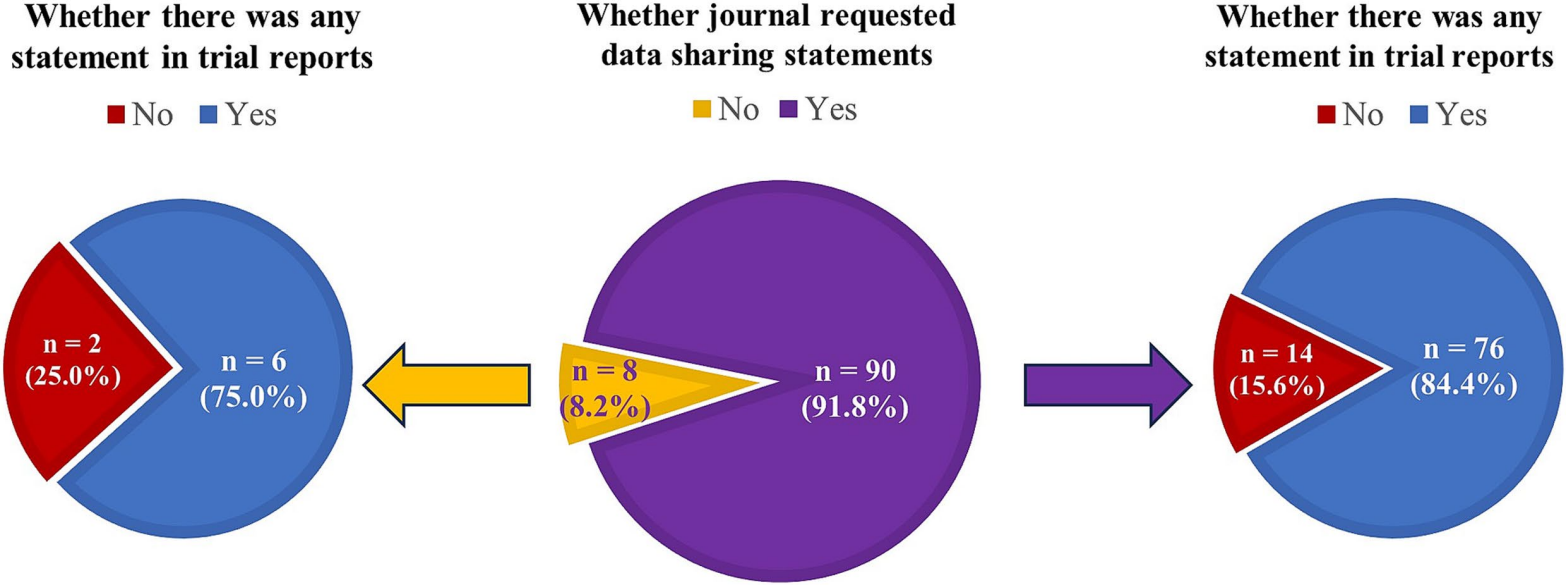
Comparison between journal request identified from submission instructions and publication of data sharing statements in clinical trial reports.

A total of 98 journals that published clinical trial reports from December 2023 to May 2024 were identified, among which 90 (91.8%) journals requested DSS and 8 (8.2%) did not (Supplementary Tables 1, 3, 4). For the journals requesting DSS, 14 (15.6%) did not publish any DSS in their trial reports published between December 2023 and May 2024 (Figure 4 and Supplementary Table 4). For the remaining eight journals without DSS request, 2 (25.0%) did not include any DSS in their trial reports. However, the discordance between journals’ declared request for DSS and their actual publication of DSS was not significant (*p* = 0.12).

Among the 90 journals requesting DSS from submission instructions, journals publishing DSS in trial reports was found to publish more trials, have a lower JCR quartile and higher impact factor, when compared with journals that did not publish DSS (Supplementary Figure 4). There were 39 and 51 journals having weak and strong request, respectively. Specifically, 25.6% (10/39) of journals with weak request and 7.8% (4/51) of journals with strong request did not publish any DSS in their trial reports (Supplementary Table 4). No significant difference in the proportions of journals publishing DSS was observed among the three journals groups (having no request, weak request, and strong request; *p* = 0.06). Moreover, when separated by these three journal groups, no significant differences in characteristics were detected in journals publishing DSS when compared with those that did not publish DSS in trial reports (Supplementary Table 5).

## Discussion

In this study, we systematically investigated the current practice of endocrinology and metabolism journals regarding their requesting and publishing DSS in clinical trials. The principal findings were as follows: (i) there were over 10% of journals that did not request DSS in trial submissions; (ii) among the journals requesting DSS, more than 15% indeed did not publish any DSS in their published trial reports.

Some prior studies had explored the practice of journals requesting DSS for clinical trials. For instance, one study including 120 journals with the highest impact factor across three disciplines (neuroscience, physics, operations research) in 2019, reported the proportions of journals requesting DSS ranging from 38 to 60% (12). Other studies that focused on different fields by including different amounts of journals, also found a varying journal request proportion from 30 to 79% (13–19). While none of previous studies specifically evaluated endocrinology and metabolism journals, our survey showed the majority of journals (89%) requested DSS in clinical trial submissions, indicating a relatively good practice of DSS request for trial reports. This positive result may be partly due to the rising concern over the increasing burden of metabolic diseases and the elevated awareness of requiring reproducible evidence in this area (27). Nevertheless, there remained 11% of the journals without DSS request, which revealed a room towards enhancing inclusion of DSS and thus reporting transparency of clinical trials in endocrinology and metabolism journals.

To the best of our knowledge, this study was the first to systematically explore journal request for DSS in endocrinology and metabolism journals. For instance, there were only seven and three endocrinology and metabolism journals included in two previous studies for assessing journal request, respectively (13, 14). By matching their journals with ours, four journals that did not request DSS in 2018 were observed to change to requesting DSS in 2023, which might reflect a temporal improvement given the evolving impact of ICMJE requirement. Notably, findings from these matched data should be interpreted with caution, given the small sample size and potential selection bias of journals from the two previous studies.

Journals requesting DSS had a significantly higher impact factor, lower JCR quartile, and published more trials from 2019 to 2022. In general, rigorous peer review procedures and increased publication quality were found in journals having a higher impact factor and publishing more trial reports (28). Of note, 50% (8/16) of journals without DSS request were on the ICMJE list (Supplementary Table 2). This was contrary to their commitment to endorse ICMJE requirement for enhancing the quality of medical science and its reporting. Although the ICMJE noted that “there may be some listed journals that do not follow all of the many recommendations and policies in the document,” more endeavors would be needed to help advance the journal request in ICMJE-listing journals for DSS in clinical trials (16).

As a DSS specifies whether the data supporting the research could be shared and how it could be accessed, the statement has become increasingly recognized to enhance research quality and reproducibility, and promote the actual data sharing (8, 10, 11). For instance, Colavizza et al. (8) reported that among studies published by PLOS (Public Library of Science) and BMC (BioMed Central), those providing a DSS with a repository link were found to receive over 25% higher citations on average. Therefore, further efforts are required to improve the request for DSS in endocrinology and metabolism journals, and ultimately enhance the data sharing practice in this field.

Even though journal request for DSS was considered as the first critical step towards actual data sharing, journals’ subsequently published trial reports were observed to inadequately comply with their declared request for DSS from submission instructions in our study. This indicated an important deficiency and inconsistency between journals’ declared request and their actual implementation of DSS request. Likewise, Siebert et al. (14) reported that only 25% of trial reports were published with a DSS from 38 ICMJE-affiliated journals. Another study based on top surgical journals observed no change in the presence of DSS in trial reports before and after their implementation of ICMJE requirement (20). Thus, more action is needed to improve the actual implementation of journal request for DSS. Interestingly, we found 75% (six out of eight) of journals without request on their submission instructions did publish trial reports with a DSS. Trial authors are becoming more aware of the importance of DSS in their submission for improving research integrity, transparency, reproducibility and collaboration; therefore, they may be willing to provide a DSS even though it was not requested by the journal.

To enhance the request and implementation of DSS, joint endeavors are needed across all research stakeholders. Publishers and journals may enforce automated DSS verification during submission, with editors assessing the adequacy of DSS provided in submitted manuscripts during the peer review process. Researchers are encouraged to develop comprehensive data management protocols during study design and execution. Funders may mandate data sharing plans and provide user-friendly deposition platforms. The coordinated efforts could transform DSS adoption to a routine practice, ultimately improving research transparency and scientific progress.

### Strengths and limitations

We systematically surveyed the current practice of endocrinology and metabolism journals in requesting and publishing DSS, thereby generating some new insights into improving reporting transparency and eventually the actual data sharing in clinical trials. Several limitations need to be noted. First, we could not perform multivariable analysis to control potential confounding due to the small sample size of journals. Similarly, we could not conduct statistical test for the change in difference in the proportion of journal request over time, because there were two zero-value cells in the contingency table. Results from this observational study should be interpreted with caution because potential biases and confounding could not be fully precluded. No data on publishers’ policies or editors’ perspectives were collected in this survey, restricting our further in-depth exploration. Although we aimed to include all eligible endocrinology and metabolism journals, some general journals that published endocrinology and metabolism trials (for instance, *the New England Journal of Medicine,* and *the Lancet*) were not grouped as “Endocrinology & Metabolism” defined by JCR and thus not included for our analysis. Our study design included two distinct time windows for journal assessment (2019–2022 and December 2023–May 2024), which could not capture trial publication patterns during the 11-month gap (January–November 2023). Thus, these findings should be interpreted with caution and they could only reflect the practice of journals’ DSS request during our study period. Because the journals that included trials in their scope but indeed did not publish trials between 2019 and 2022 were excluded from analysis, little was known about their DSS implementation. Furthermore, our data extraction covered a 6-month period for the subsequent publications of trials (December 2023–May 2024). While this extended timeframe allowed for comprehensive manual review, it was possible that some journals updated their submission instructions during this period. Given the dynamic change in journals’ request for DSS, future investigations are required to evaluate the most recent practice of their declared and actual request for DSS.

## Conclusion

Over 10% of endocrinology and metabolism journals did not request DSS in clinical trial submissions. More than 15% of the journals declaring to request DSS from their submission instructions, did not publish DSS in their published trial reports. More efforts are needed to improve the practice of endocrinology and metabolism journals in requesting and publishing DSS of clinical trial reports.

## Data Availability

The raw data supporting the conclusions of this article will be made available by the authors, without undue reservation.
