# Supplementary material for: Request of endocrinology and metabolism journals for data sharing statements in clinical trial reports: a survey
Source: Front Med (Lausanne). 2025 May 21;12:1518399. doi: 10.3389/fmed.2025.1518399 (PMC12133921; doi:10.3389/fmed.2025.1518399)
Supplement: Supplementary file 1 [file Data_Sheet_1.docx]

**List of contents**

[**STable 1**. List of the 141 included endocrinology and metabolism journals and the 98 journals that published clinical trial reports from Dec 2023 to May 2024 2](#_Toc195625848)

[**STable 2**. Comparisons of journal characteristics among journals having no, weak or strong request for DSS* 7](#_Toc195625849)

[**STable 3**. Descriptions of journal characteristics for the 98 journals that published clinical trial reports from Dec 2023 to May 2024 9](#_Toc195625850)

[**STable 4**. Publication of data sharing statements in clinical trial reports versus the journal request for data sharing statements identified from submission instructions among all journals (A) and by request strength (B) 11](#_Toc195625851)

[**STable 5.** Comparisons of journal characteristics regarding publication of data sharing statements in clinical trial reports among journals with no, weak or strong request for DSS*^#^ 12](#_Toc195625852)

[**SFigure 1**. Timeframe of the study 15](#_Toc195625853)

[**SFigure 2**. The included journals from different publishers listed in descending order by frequency 16](#_Toc195625854)

[**SFigure 3**. The included journals from different regions of the institution of editor-in chief listed in descending order by frequency 17](#_Toc195625855)

[**SFigure 4.** Comparison between journals with and without publishing data sharing statements in their published trial reports among those journals requesting statements from submission instructions (n = 90)^*^ 18](#_Toc195625856)

# **STable 1**. List of the 141 included endocrinology and metabolism journals and the 98 journals that published clinical trial reports from Dec 2023 to May 2024

| **Journals** | **Whether a journal explicitly requested a data sharing statement on its manuscript submission instructions^1^** | **Whether a journal published any data sharing statement in its clinical trial reports** |
| --- | --- | --- |
| Acta Diabetologica | Yes-Weak | No |
| Adipocyte | Yes-Strong | -* |
| Aging Male | Yes-Weak | Yes |
| American Journal of Physiology-Endocrinology and Metabolism | Yes-Strong | Yes |
| Annales D Endocrinologie | No request | -* |
| Annals of Nutrition and Metabolism | Yes-Strong | Yes |
| Antioxidants & Redox Signaling | Yes-Weak | -* |
| Archives of Endocrinology Metabolism | Yes-Strong | -* |
| Archives of Osteoporosis | Yes-Weak | -* |
| Archives of Physiology and Biochemistry | Yes-Weak | Yes |
| Biofactors | Yes-Strong | -* |
| Biological Trace Element Research | Yes-Strong | Yes |
| Biology of Sex Differences | Yes-Strong | -* |
| Bmc Endocrine Disorders | Yes-Strong | Yes |
| Bmj Open Diabetes Research & Care | Yes-Strong | Yes |
| Bone | Yes-Strong | Yes |
| Bone Reports | Yes-Weak | Yes |
| Calcified Tissue International | Yes-Weak | Yes |
| Canadian Journal of Diabetes | Yes-Weak | No |
| Cardiovascular Diabetology | Yes-Strong | Yes |
| Cell Metabolism | Yes-Strong | -* |
| Clinical Diabetology | Yes-Weak | Yes |
| Clinical Endocrinology | Yes-Strong | Yes |
| Clinical Medicine Insights-Endocrinology and Diabetes | Yes-Weak | -* |
| Clinical Obesity | Yes-Strong | Yes |
| Clinical Pediatric Endocrinology | No request | Yes |
| Current Diabetes Reports | Yes-Strong | -* |
| Current Diabetes Reviews | Yes-Strong | -* |
| Diabetes | Yes-Strong | Yes |
| Diabetes & Metabolic Syndrome-Clinical Research & Reviews | Yes-Weak | Yes |
| Diabetes & Metabolism | Yes-Weak | Yes |
| Diabetes & Metabolism Journal | Yes-Strong | No |
| Diabetes & Vascular Disease Research | Yes-Weak | Yes |
| Diabetes Care | Yes-Strong | Yes |
| Diabetes Metabolic Syndrome and Obesity-Target & Therapy | Yes-Strong | Yes |
| Diabetes Obesity & Metabolism | Yes-Strong | Yes |
| Diabetes Research and Clinical Practice | Yes-Weak | Yes |
| Diabetes Technology & Therapeutics | Yes-Weak | No |
| Diabetes Therapy | Yes-Strong | Yes |
| Diabetes-Metabolism Research and Reviews | Yes-Strong | Yes |
| Diabetic Medicine | Yes-Strong | Yes |
| Diabetologia | Yes-Strong | Yes |
| Diabetology & Metabolic Syndrome | Yes-Strong | Yes |
| Diabetology International | Yes-Strong | Yes |
| Discover Oncology | Yes-Strong | -* |
| Endocrine | Yes-Weak | Yes |
| Endocrine Connections | Yes-Strong | No |
| Endocrine Journal | Yes-Strong | -* |
| Endocrine Metabolic & Immune Disorders-Drug Targets | Yes-Strong | Yes |
| Endocrine Practice | Yes-Weak | Yes |
| Endocrine Research | Yes-Weak | -* |
| Endocrine-Related Cancer | Yes-Strong | -* |
| Endocrinologia Diabetes Y Nutricion | Yes-Weak | -* |
| Endocrinology | Yes-Strong | -* |
| Endocrinology and Metabolism | Yes-Weak | No |
| Endocrinology Diabetes & Metabolism | Yes-Strong | No |
| Endokrynologia Polska | No request | -* |
| European Journal of Endocrinology | Yes-Weak | Yes |
| European Thyroid Journal | Yes-Strong | Yes |
| Free Radical Biology and Medicine | Yes-Strong | Yes |
| Frontiers In Endocrinology | Yes-Strong | Yes |
| Growth Factors | Yes-Weak | Yes |
| Growth Hormone & Igf Research | Yes-Weak | -* |
| Gynecological Endocrinology | Yes-Strong | Yes |
| Hormone and Metabolic Research | No request | No |
| Hormone Research in Paediatrics | Yes-Strong | -* |
| Hormones and Behavior | Yes-Weak | Yes |
| Hormones-International Journal of Endocrinology and Metabolism | Yes-Weak | Yes |
| International Journal of Diabetes in Developing Countries | Yes-Weak | Yes |
| International Journal of Endocrinology | Yes-Weak | -* |
| International Journal of Endocrinology and Metabolism | No request | Yes |
| International Journal of Obesity | Yes-Strong | Yes |
| Jbmr Plus | Yes-Strong | Yes |
| Journal of Biological Regulators and Homeostatic Agents | Yes-Strong | Yes |
| Journal of Bone and Mineral Metabolism | No request | Yes |
| Journal of Bone and Mineral Research | Yes-Strong | Yes |
| Journal of Cerebral Blood Flow and Metabolism | Yes-Strong | Yes |
| Journal of Clinical Densitometry | Yes-Weak | No |
| Journal of Clinical Endocrinology & Metabolism | Yes-Strong | Yes |
| Journal of Clinical Research In Pediatric Endocrinology | No request | -* |
| Journal of Diabetes | Yes-Strong | Yes |
| Journal of Diabetes and Its Complications | Yes-Weak | Yes |
| Journal of Diabetes and Metabolic Disorders | No request | Yes |
| Journal of Diabetes Investigation | No request | Yes |
| Journal of Diabetes Research | Yes-Strong | Yes |
| Journal of Diabetes Science and Technology | Yes-Weak | Yes |
| Journal of Diabetology | No request | No |
| Journal of Endocrinological Investigation | Yes-Weak | Yes |
| Journal of Endocrinology and Metabolism | Yes-Strong | -* |
| Journal of Endocrinology Metabolism and Diabetes of South Africa | No request | -* |
| Journal of Inherited Metabolic Disease | Yes-Strong | Yes |
| Journal of Neuroendocrinology | Yes-Strong | Yes |
| Journal of Obesity | Yes-Strong | -* |
| Journal of Obesity & Metabolic Syndrome | Yes-Weak | No |
| Journal of Pediatric Endocrinology & Metabolism | No request | Yes |
| Journal of Pineal Research | Yes-Strong | Yes |
| Journal of Steroid Biochemistry and Molecular Biology | Yes-Weak | -* |
| Journal of The ASEAN Federation of Endocrine Societies | Yes-Strong | -* |
| Journal of The Endocrine Society | Yes-Strong | Yes |
| Journal of Thyroid Research | Yes-Weak | Yes |
| Journal of Trace Elements in Medicine and Biology | Yes-Weak | No |
| Lancet Diabetes & Endocrinology | Yes-Strong | Yes |
| Magnesium Research | Yes-Strong | -* |
| Metabolic Brain Disease | Yes-Strong | -* |
| Metabolism-Clinical and Experimental | Yes-Weak | Yes |
| Metabolomics | Yes-Strong | Yes |
| Minerva Endocrinology | Yes-Weak | -* |
| Molecular and Cellular Endocrinology | Yes-Strong | -* |
| Molecular Genetics and Metabolism | Yes-Weak | Yes |
| Molecular Metabolism | Yes-Strong | Yes |
| Nature Metabolism | Yes-Strong | Yes |
| Neuroendocrinology | Yes-Strong | -* |
| Neuroendocrinology Letters | No request | -* |
| Neuroimmunomodulation | No request | -* |
| Neuropeptides | Yes-Weak | -* |
| Nutrition & Diabetes | Yes-Strong | Yes |
| Nutrition Clinique Et Metabolisme | No request | -* |
| Nutrition Metabolism and Cardiovascular Diseases | Yes-Weak | Yes |
| Obesity | Yes-Strong | Yes |
| Obesity Facts | Yes-Strong | Yes |
| Obesity Research & Clinical Practice | Yes-Weak | Yes |
| Obesity Science & Practice | Yes-Strong | No |
| Osteoporosis and Sarcopenia | Yes-Strong | -* |
| Osteoporosis International | Yes-Weak | Yes |
| Pediatric Diabetes | Yes-Strong | Yes |
| Peptides | Yes-Weak | -* |
| Pituitary | Yes-Weak | -* |
| Primary Care Diabetes | Yes-Weak | Yes |
| Prostaglandins Leukotrienes and Essential Fatty Acids | Yes-Weak | Yes |
| Prostate | Yes-Strong | Yes |
| Psychoneuroendocrinology | Yes-Weak | Yes |
| Reproductive Biology and Endocrinology | Yes-Strong | Yes |
| Reviews In Endocrine & Metabolic Disorders | Yes-Strong | -* |
| Science of Diabetes Self-Management and Care | Yes-Weak | No |
| Steroids | Yes-Weak | No |
| Stress-The International Journal on The Biology of Stress | Yes-Weak | No |
| Therapeutic Advances in Endocrinology and Metabolism | Yes-Strong | -* |
| Thyroid | Yes-Weak | Yes |
| Thyroid Research | Yes-Strong | -* |
| Turkish Journal of Endocrinology and Metabolism | No request | -* |
| World Journal of Diabetes | Yes-Strong | Yes |

^1^ The strength of journal request was deemed as Weak by using the terms “encourage” or “recommend” (e.g., Authors are encouraged to provide a Data Sharing Statement.); if a journal used the terms “mandate”, “must”, “require”, “request” or “should” (e.g., All research articles should include a Data Sharing Statement), the strength of journal request was deemed as Strong.

^*^ Journal that did not publish any trial report between Dec 2023 and May 2024 were excluded from analysis regarding assessment of DSS publication in clinical trial reports (n = 43)

# **STable 2**. Comparisons of journal characteristics among journals having no, weak or strong request for DSS*

| **Journal characteristics** | **Overall (n = 141)** | **No request (n = 16)** | **Weak request (n = 51)** | **Strong request (n = 74)** | ***P*-value** |
| --- | --- | --- | --- | --- | --- |
| **Open Access percentage:** Median (Q1 - Q3) | 24.5 (11.1 - 93.5) | 45.9 (6.7 - 96.5) | 19.3 (9.1 - 27.4) | 34.2 (16.5 - 98.0) | 0.01 |
| **Open Access percentage ≥ 50%** |  |  |  |  |  |
| No | 92 (65.2) | 8 (50.0) | 43 (84.3) | 41 (55.4) | 0.002 |
| Yes | 49 (34.8) | 8 (50.0) | 8 (15.7) | 33 (44.6) |  |
| **Publisher** |  |  |  |  |  |
| Elsevier | 26 (18.4) | 0 (0) | 20 (39.2) | 6 (8.1) | <0.001 |
| Springer | 21 (14.9) | 2 (12.5) | 9 (17.6) | 10 (13.5) |  |
| Wiley | 18 (12.8) | 1 (6.3) | 0 (0) | 17 (23.0) |  |
| Others | 76 (53.9) | 13 (81.3) | 22 (43.1) | 41 (55.4) |  |
| **Publication language** |  |  |  |  |  |
| English | 139 (98.6) | 14 (87.5) | 51 (100) | 74 (100) | - |
| Non-English | 2 (1.4) | 2 (12.5) | 0 (0) | 0 (0) |  |
| **Journal impact factor:** Median (Q1 - Q3) | 3.5 (2.5 - 4.9) | 2.0 (0.7 - 2.5) | 3.4 (2.6 - 4.2) | 4.1 (3.2 - 5.9) | <0.001 |
| **JIF ≥3.5^1^** |  |  |  |  |  |
| No | 68 (48.2) | 16 (100) | 26 (51.0) | 26 (35.1) | <0.001 |
| Yes | 73 (51.8) | 0 (0) | 25 (49.0) | 48 (64.9) |  |
| **JCR Quartile** |  |  |  |  |  |
| Q1~2 | 67 (47.5) | 0 (0) | 21 (41.2) | 46 (62.2) | <0.001 |
| Q3~4 | 74 (52.5) | 16 (100) | 30 (58.8) | 28 (37.8) |  |
| **Whether the journal was on the ICMJE list** |  |  |  |  |  |
| No | 96 (68.1) | 8 (50.0) | 40 (78.4) | 48 (64.9) | 0.07 |
| Yes | 45 (31.9) | 8 (50.0) | 11 (21.6) | 26 (35.1) |  |
| **Whether the journal endorsed CONSORT** |  |  |  |  |  |
| No | 59 (41.8) | 8 (50.0) | 29 (56.9) | 22 (29.7) | 0.01 |
| Yes | 82 (58.2) | 8 (50.0) | 22 (43.1) | 52 (70.3) |  |
| **Number of trials published between 2019 and 2022:** Median (Q1 - Q3) | 18.0 (5.0 – 38.0) | 4.5 (2.8 - 20.8) | 20.0 (7.5 - 33.0) | 20.0 (6.0 - 51.5) | 0.07 |
| **Number of trials published between 2019 and 2022 ≥ 18^1^** |  |  |  |  |  |
| No | 70 (49.6) | 12 (75.0) | 24 (47.1) | 34 (45.9) | 0.10 |
| Yes | 71 (50.4) | 4 (25.0) | 27 (52.9) | 40 (54.1) |  |
| **Gender of Editor-in chief** |  |  |  |  |  |
| Female | 28 (19.9) | 2 (12.5) | 9 (17.6) | 17 (23.0) | 0.56 |
| Male | 113 (80.1) | 14 (87.5) | 42 (82.4) | 57 (77.0) |  |
| **Region of the institution of Editor-in chief** |  |  |  |  |  |
| USA | 46 (32.6) | 1 (6.3) | 16 (31.4) | 29 (39.2) | 0.003 |
| UK | 18 (12.8) | 0 (0) | 5 (9.8) | 13 (17.6) |  |
| Italy | 15 (10.6) | 0 (0) | 8 (15.7) | 7 (9.5) |  |
| Germany | 10 (7.1) | 3 (18.8) | 2 (3.9) | 5 (6.8) |  |
| Others | 52 (36.9) | 12 (75.0) | 20 (39.2) | 20 (27.0) |  |

* Results shown as count (%) unless otherwise specified

^1^ The median Journal Impact Factor was 3.5; median number of trials published between 2019 and 2022 was 18

Note: DSS (data sharing statement); JCR (Journal Citation Reports); ICMJE (International Committee of Medical Journal Editors); CONSORT (Consolidated Standards of Reporting Trials); Q1 (first quartile); Q3 (third quartile)

# **STable 3**. Descriptions of journal characteristics for the 98 journals that published clinical trial reports from Dec 2023 to May 2024

| **Journal characteristics** | **Overall (n = 98)** |
| --- | --- |
| **Open Access percentage:** Median (Q1 - Q3) | 24.5 (11.1 - 78.3) |
| **Open Access percentage ≥ 50%** |  |
| No | 67 (68.4) |
| Yes | 31 (31.6) |
| **Publisher** |  |
| Elsevier | 21 (21.4) |
| Springer | 16 (16.3) |
| Wiley | 17 (17.3) |
| Others | 44 (44.9) |
| **Publication language** |  |
| English | 98 (100) |
| **Journal impact factor:** Median (Q1 - Q3) | 3.8 (2.8 - 5.2) |
| **JCR Quartile** |  |
| Q1~2 | 51 (52.0) |
| Q3~4 | 47 (48.0) |
| **Whether the journal was on the ICMJE list** |  |
| No | 66 (67.3) |
| Yes | 32 (32.7) |
| **Whether the journal endorsed CONSORT** |  |
| No | 39 (39.8) |
| Yes | 59 (60.2) |
| **Number of trials published between 2019 and 2022:** Median (Q1 - Q3) | 28.0 (12.0 - 56.8) |
| **Gender of Editor-in chief** |  |
| Female | 16 (16.3) |
| Male | 82 (83.7) |
| **Region of the institution of Editor-in chief** |  |
| USA | 34 (34.7) |
| UK | 16 (16.3) |
| Italy | 10 (10.2) |
| Germany | 7 (7.1) |
| Others | 31 (31.6) |

* Results shown as count (%) unless otherwise specified

Note: JCR (Journal Citation Reports); ICMJE (International Committee of Medical Journal Editors); CONSORT (Consolidated Standards of Reporting Trials); Q1 (first quartile); Q3 (third quartile)

# **STable 4**. Publication of data sharing statements in clinical trial reports versus the journal request for data sharing statements identified from submission instructions among all journals (A) and by request strength (B)

1. Publication of data sharing statements in clinical trial reports versus journal request for data sharing statements among all journals*

|  | | **Journal request for data sharing statements from submission instructions** | | **Total** |
| --- | --- | --- | --- | --- |
|  |  | No | Yes |  |
| **Whether there was any data sharing statement in clinical trial reports published in the journal** | None | 2 (2.1) | 14 (14.2) | 16 (16.3) |
|  | Any | 6 (6.1) | 76 (77.6) | 82 (83.7) |
| **Total** | | 8 (8.2) | 90 (91.8) | 98 (100) |

* Only 98 journals that published clinical trial reports from Dec 1^st^, 2023 to May 31^st^, 2024 were included; results shown as count (%)

1. Publication of data sharing statements in clinical trial reports versus journal request for data sharing statements by request strength*

|  | | **Strength of journal request for data sharing statements from submission instructions** | | **Total** |
| --- | --- | --- | --- | --- |
|  |  | Weak | Strong |  |
| **Whether there was any data sharing statement in clinical trial reports published in the journal** | None | 10 (11.1) | 4 (4.4) | 14 (15.6) |
|  | Any | 29 (32.2) | 47 (52.2) | 76 (74.4) |
| **Total** | | 39 (43.3) | 51 (56.7) | 90 (100) |

* Only 90 journals requesting data sharing statements that published clinical trial reports from Dec 1^st^, 2023 to May 31^st^, 2024 were included; results shown as count (%)

# **STable 5.** Comparisons of journal characteristics regarding publication of data sharing statements in clinical trial reports among journals with no, weak or strong request for DSS*^#^

| **Journal characteristics** | **No request** | | | | **Weak request** | | | | **Strong request** | | | |
| --- | --- | --- | --- | --- | --- | --- | --- | --- | --- | --- | --- | --- |
|  | **Overall (n = 8)** | **Publication of data sharing statements** | | ***P*-value** | **Overall (n = 39)** | **Publication of data sharing statements** | | ***P*-value** | **Overall (n = 51)** | **Publication of data sharing statements** | | ***P*-value** |
|  |  | **No  (n = 2)** | **Yes  (n = 6)** |  |  | **No (n = 10)** | **Yes  (n = 29)** |  |  | **No  (n = 4)** | **yes (n = 47)** |  |
| **Open Access percentage:** Median (Q1 - Q3) | 34.3 (6.5 - 80.8) | 52.9 (30.4 - 75.4) | 33.7 (6.0 - 71.5) | 0.64 | 19.7 (9.8 - 27.4) | 17.4 (8.6 - 27.9) | 19.7 (10.4 - 27.2) | >0.99 | 30.3 (16.8 - 96.8) | 83.3 (74.5 - 93.8) | 28.8 (15.8 - 96.8) | 0.10 |
| **Open Access percentage ≥ 50%** |  |  |  |  |  |  |  |  |  |  |  |  |
| No | 4 (50.0) | 1 (50.0) | 3 (50.0) | >0.99 | 33 (84.6) | 8 (80.0) | 25 (86.2) | >0.99 | 30 (58.8) | - | 30 (63.8) | 0.05 |
| Yes | 4 (50.0) | 1 (50.0) | 3 (50.0) |  | 6 (15.4) | 2 (20.0) | 4 (13.8) |  | 21 (41.2) | 4 (100) | 17 (36.2) |  |
| **Publisher** |  |  |  |  |  |  |  |  |  |  |  |  |
| Elsevier | - | - | - | >0.99 | 17 (43.6) | 4 (40.0) | 13 (44.8) | 0.71 | 4 (7.8) | - | 4 (8.5) | >0.99 |
| Springer | 2 (25.0) | - | 2 (33.3) |  | 7 (17.9) | 1 (10.0) | 6 (20.7) |  | 7 (13.7) | - | 7 (14.9) |  |
| Wiley | 1 (12.5) | - | 1 (16.7) |  | - | - | - |  | 16 (31.4) | 2 (50.0) | 14 (29.8) |  |
| Others | 5 (62.5) | 2 (100) | 3 (50.0) |  | 15 (38.5) | 5 (50.0) | 10 (34.5) |  | 24 (47.1) | 2 (50.0) | 22 (46.8) |  |
| **Journal impact factor:** Median (Q1 - Q3) | 2.2 (1.4 - 2.9) | 1.3 (0.8 - 1.7) | 2.5 (1.6 - 3.1) | 0.40 | 3.5 (2.6 - 4.7) | 3.1 (2.5 - 3.7) | 3.7 (2.9 - 5.0) | 0.29 | 4.2 (3.4 - 6.2) | 2.7 (2.4 - 3.7) | 4.3 (3.6 - 6.3) | 0.08 |
| **Journal impact factor ≥ 3.5** |  |  |  |  |  |  |  |  |  |  |  |  |
| No | 8 (100) | 2 (100) | 6 (100) | - | 18 (46.2) | 6 (60.0) | 12 (41.4) | 0.52 | 14 (27.5) | 3 (75.0) | 11 (23.4) | 0.10 |
| Yes | **-** | - | - |  | 21 (53.8) | 4 (40.0) | 17 (58.6) |  | 37 (72.5) | 1 (25.0) | 36 (76.6) |  |
| **JCR Quartile** |  |  |  |  |  |  |  |  |  |  |  |  |
| Q1~2 | **-** | - | - | - | 17 (43.6) | 3 (30.0) | 14 (48.3) | 0.53 | 34 (66.7) | 1 (25.0) | 33 (70.2) | 0.19 |
| Q3~4 | 8 (100) | 2 (100) | 6 (100) |  | 22 (56.4) | 7 (70.0) | 15 (51.7) |  | 17 (33.3) | 3 (75.0) | 14 (29.8) |  |
| **Whether the journal was on the ICMJE list** |  |  |  |  |  |  |  |  |  |  |  |  |
| No | 4 (50.0) | - | 4 (66.7) | 0.41 | 30 (76.9) | 8 (80.0) | 22 (75.9) | >0.99 | 32 (62.7) | 3 (75.0) | 29 (61.7) | >0.99 |
| Yes | 4 (50.0) | 2 (100) | 2 (33.3) |  | 9 (23.1) | 2 (20.0) | 7 (24.1) |  | 19 (37.3) | 1 (25.0) | 18 (38.3) |  |
| **Whether the journal endorsed CONSORT** |  |  |  |  |  |  |  |  |  |  |  |  |
| No | 4 (50.0) | 1 (50.0) | 3 (50.0) | >0.99 | 22 (56.4) | 5 (50.0) | 17 (58.6) | 0.92 | 13 (25.5) | 1 (25.0) | 12 (25.5) | >0.99 |
| Yes | 4 (50.0) | 1 (50.0) | 3 (50.0) |  | 17 (43.6) | 5 (50.0) | 12 (41.4) |  | 38 (74.5) | 3 (75.0) | 35 (74.5) |  |
| **Number of trials published between 2019 and 2022:** Median (Q1 - Q3) | 24.5 (5.0 - 53.0) | 11.0 (8.0 - 14.0) | 37.0 (11.8 - 75.0) | 0.50 | 25.0 (11.5 - 42.5) | 12.5 (6.5 - 25.8) | 28.0 (17.0 - 50.0) | 0.12 | 35.0 (17.0 - 74.5) | 15.5 (10.0 - 20.5) | 38.0 (17.5 - 76.5) | 0.08 |
| **Number of trials published between 2019 and 2022 ≥ 18** |  |  |  |  |  |  |  |  |  |  |  |  |
| No | 4 (50.0) | 2 (100) | 2 (33.3) | 0.41 | 14 (35.9) | 6 (60.0) | 8 (27.6) | 0.14 | 14 (27.5) | 2 (50.0) | 12 (25.5) | 0.64 |
| Yes | 4 (50.0) | - | 4 (66.7) |  | 25 (64.1) | 4 (40.0) | 21 (72.4) |  | 37 (72.5) | 2 (50.0) | 35 (74.5) |  |
| **Gender of Editor-in chief** |  |  |  |  |  |  |  |  |  |  |  |  |
| Female | **-** | - | - | - | 8 (20.5) | 1 (10.0) | 7 (24.1) | 0.61 | 8 (15.7) | - | 8 (17.0) | >0.99 |
| Male | 8 (100) | 2 (100) | 6 (100) |  | 31 (79.5) | 9 (90.0) | 22 (75.9) |  | 43 (84.3) | 4 (100) | 39 (83.0) |  |
| **Region of the institution of Editor-in chief** |  |  |  |  |  |  |  |  |  |  |  |  |
| USA | **-** | - | - | 0.46 | 13 (33.3) | 6 (60.0) | 7 (24.1) | 0.36 | 21 (41.2) | 2 (50.0) | 19 (40.4) | >0.99 |
| UK | **-** | - | - |  | 3 (7.7) | - | 3 (10.3) |  | 13 (25.5) | 1 (25.0) | 12 (25.5) |  |
| Italy | **-** | - | - |  | 6 (15.4) | 1 (10.0) | 5 (17.2) |  | 4 (7.8) | - | 4 (8.5) |  |
| Germany | 2 (25.0) | 1 (50.0) | 1 (16.7) |  | 1 (2.6) | - | 1 (3.4) |  | 4 (7.8) | - | 4 (8.5) |  |
| Others | 6 (75.0) | 1 (50.0) | 5 (83.3) |  | 16 (41.0) | 3 (30.0) | 13 (44.8) |  | 9 (17.6) | 1 (25.0) | 8 (17.0) |  |

* Results shown as count (%) unless otherwise specified

^#^ Only 98 journals that published clinical trial reports from Dec 1^st^, 2023 to May 31^st^, 2024 were selected.

Note: DSS (data sharing statement); JCR (Journal Citation Reports); ICMJE (International Committee of Medical Journal Editors); CONSORT (Consolidated Standards of Reporting Trials); Q1 (first quartile); Q3 (third quartile)


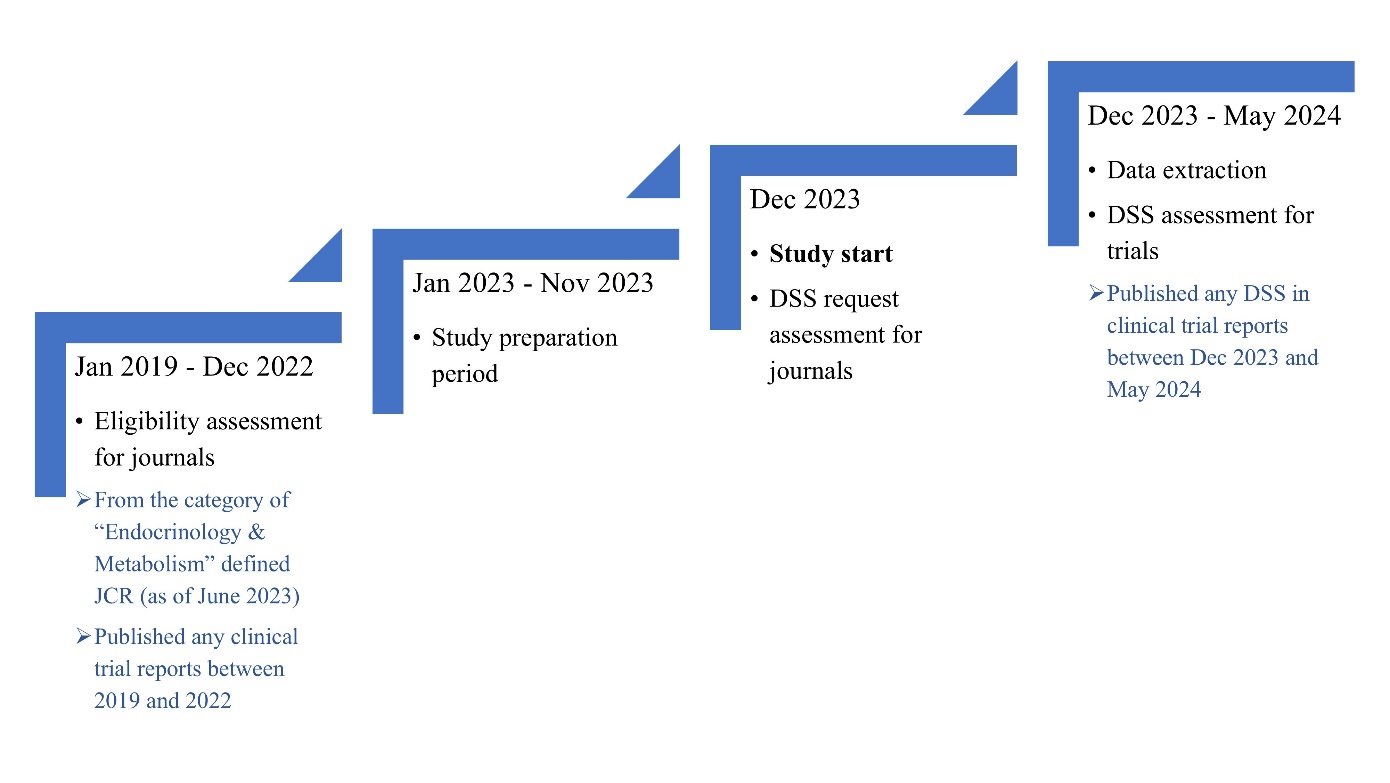


# **SFigure 1**. Timeframe of the study


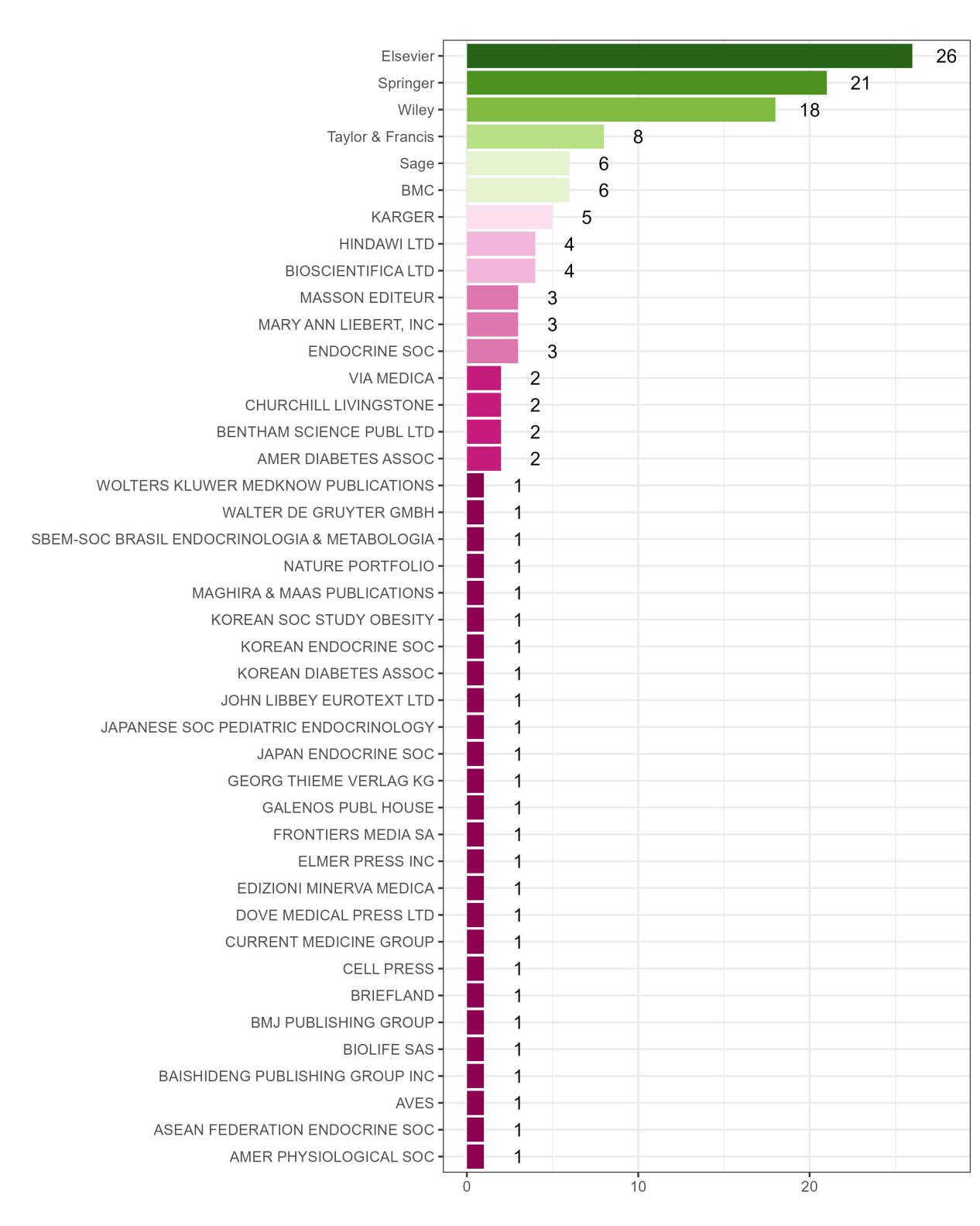


# **SFigure 2**. The included journals from different publishers listed in descending order by frequency


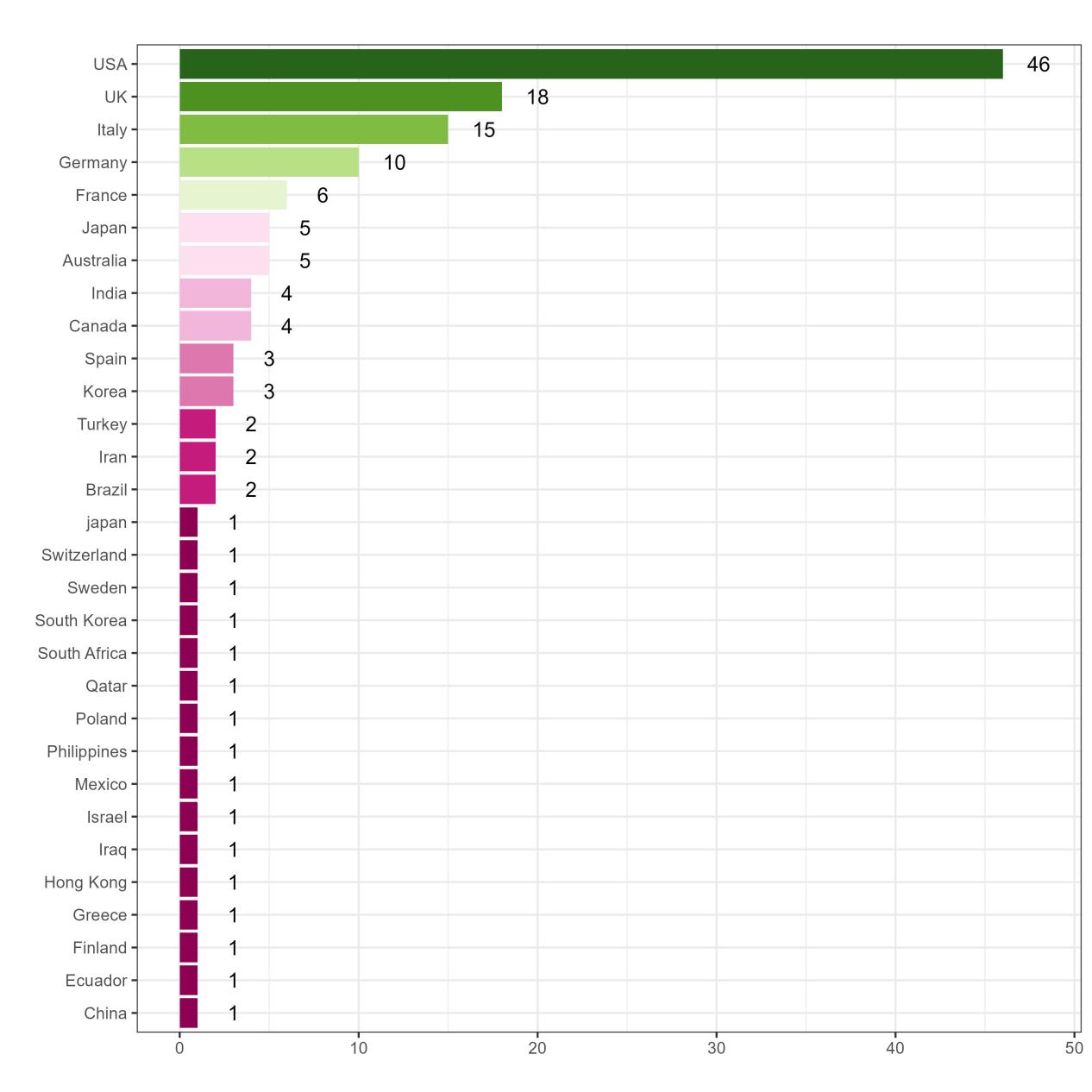


# **SFigure 3**. The included journals from different regions of the institution of editor-in chief listed in descending order by frequency


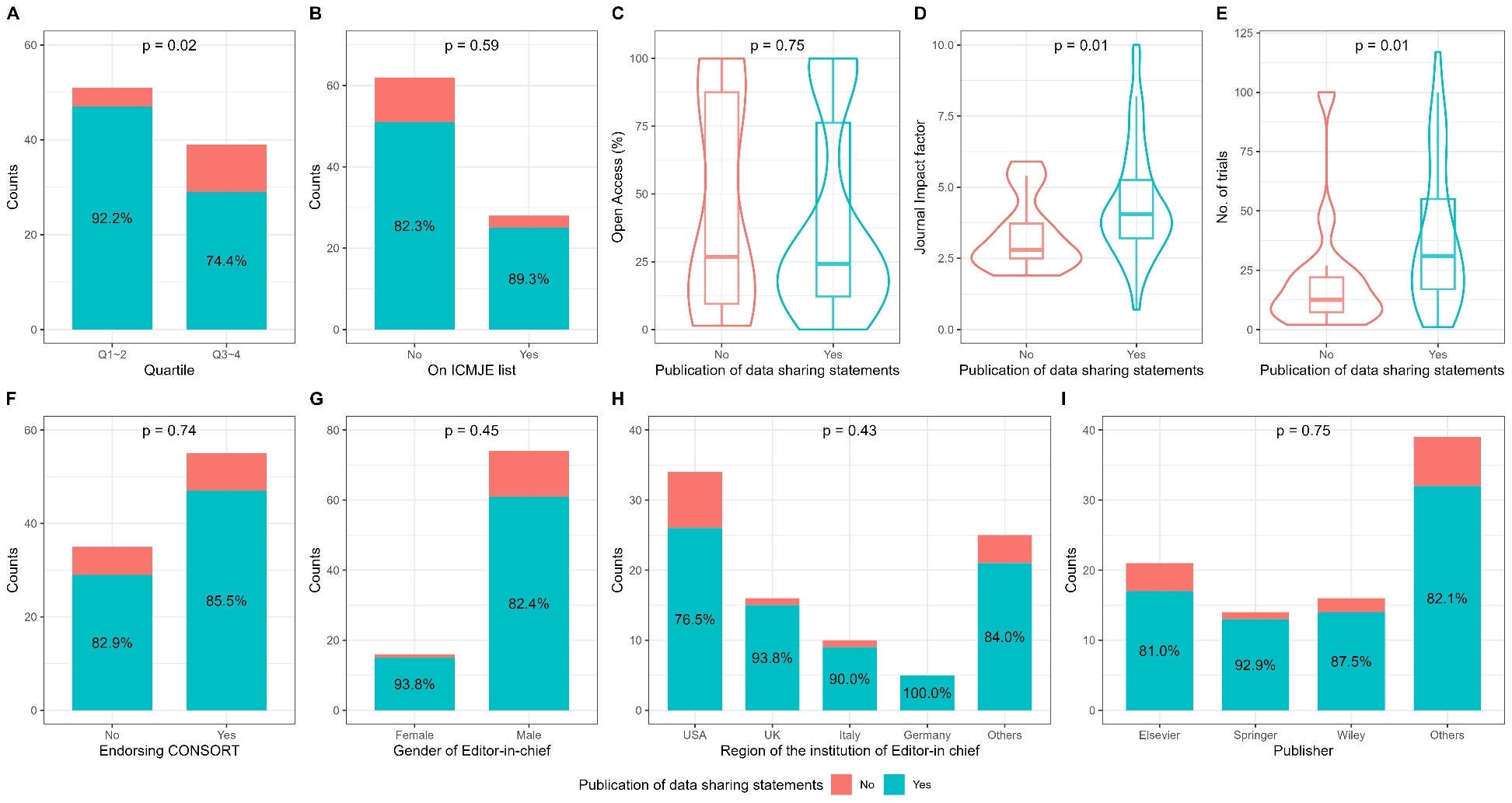


# **SFigure 4.** Comparison between journals with and without publishing data sharing statements in their published trial reports among those journals requesting statements from submission instructions (n = 90)^*^

^*^ Only 90 journals requesting data sharing statements that published clinical trial reports from Dec 1^st^, 2023 to May 31^st^, 2024 were selected.
